# Comparing Paclitaxel Plus Fluorouracil Versus Cisplatin Plus Fluorouracil in Chemoradiotherapy for Locally Advanced Esophageal Squamous Cell Cancer: A Randomized, Multicenter, Phase III Clinical Trial

**DOI:** 10.1200/JCO.18.02122

**Published:** 2019-03-28

**Authors:** Yun Chen, Jinjun Ye, Zhengfei Zhu, Weixin Zhao, Jialiang Zhou, Chaoyang Wu, Huarong Tang, Min Fan, Ling Li, Qin Lin, Yi Xia, Yunhai Li, Jiancheng Li, Huixun Jia, Saiquan Lu, Zhen Zhang, Kuaile Zhao

**Affiliations:** ^1^ Fudan University Shanghai Cancer Center, Shanghai, China; ^2^ Jiangsu Cancer Hospital, Nanjing, China; ^3^ Affiliated Hospital of Jiangnan University, Wuxi, China; ^4^ Zhenjiang First People’s Hospital, Zhenjiang, China; ^5^ The First Affiliated Hospital of Xiamen University, Xiamen, China; ^6^ Fudan University Shanghai Cancer Center Minhang Branch Hospital, Shanghai, China; ^7^ Fujian Provincial Cancer Hospital, Fuzhou, China; ^8^ Shanghai General Hospital, Shanghai, China

## Abstract

**PURPOSE:**

This trial aimed to assess the efficacy and safety of the paclitaxel plus fluorouracil regimen versus the cisplatin plus fluorouracil regimen in definitive concurrent chemoradiotherapy (dCRT) in patients with locally advanced esophageal squamous cell carcinoma (ESCC).

**PATIENTS AND METHODS:**

Patients with locally advanced ESCC were enrolled and randomly assigned to either the paclitaxel plus fluorouracil group or the cisplatin plus fluorouracil group. The patients in the paclitaxel plus fluorouracil group were treated with paclitaxel and fluorouracil one cycle per week in dCRT for five cycles followed by paclitaxel and fluorouracil one cycle per month in consolidation chemotherapy for two cycles. The patients in the cisplatin/5-fluorouracil group were treated with cisplatin and fluorouracil one cycle per month in dCRT for two cycles followed by two cycles in consolidation chemotherapy. The radiotherapy dose was 61.2 Gy delivered in 34 fractions. The primary end point was 3-year overall survival (OS).

**RESULTS:**

Four hundred thirty-six patients with ESCC in six centers were recruited at a 1:1 ratio between April 2012 and July 2015. The median follow-up of the surviving patients was 48.7 months (interquartile range, 42.6-60.9). The 3-year OS was 55.4% in the paclitaxel plus fluorouracil group and 51.8% in the cisplatin plus fluorouracil group (hazard ratio, 0.905 [95% CI, 0.698 to 1.172]; *P* = .448). The 3-year progression-free survival was also not significantly different between the paclitaxel plus fluorouracil group and the cisplatin plus fluorouracil group (43.7% *v* 45.5%, respectively; hazard ratio, 0.973 [95% CI, 0.762 to 1.243]; *P* = .828). Compared with the cisplatin plus fluorouracil group, the paclitaxel plus fluorouracil group had significantly lower incidences of acute grade 3 or higher anemia, thrombocytopenia, anorexia, nausea, vomiting, and fatigue (*P* < .05), but higher incidences of acute grade 3 or higher leukopenia, radiation dermatitis, and radiation pneumonitis (*P* < .05).

**CONCLUSION:**

The paclitaxel plus fluorouracil regimen did not significantly prolong the OS compared with the standard cisplatin plus fluorouracil regimen in dCRT in patients with locally advanced ESCC.

## INTRODUCTION

In China in 2015, esophageal cancer was the third most common cancer, with an estimated 477,900 new cases, and the fourth most common cause of cancer deaths, with an estimated 375,000 deaths.^1^ Ninety percent of these cases were squamous cell carcinoma.^2^ On the basis of the results of Radiation Therapy Oncology Group (RTOG) 8501, definitive radiotherapy concurrent with cisplatin plus fluorouracil is a standard modality for patients with inoperable, locally advanced esophageal cancer.^3^ However, the treatment toxicity and the survival outcomes of definitive concurrent chemoradiotherapy (dCRT) with cisplatin plus fluorouracil regimen were not satisfactory, with 42% grade 3 acute toxicities, 25% grade 3 late toxicities, and 26% 5-year overall survival (OS).^3^

Paclitaxel showed a considerable efficiency in metastatic esophageal cancer in clinical studies and was a radiation sensitizer in preclinical studies.^4-6^ Paclitaxel-based chemoradiotherapy regimens had been investigated in phase I and II studies for neoadjuvant concurrent chemoradiotherapy and dCRT in patients with esophageal cancer, and they showed promising results.^5,7-9^ Although there were differences between these studies in terms of the paclitaxel dose and combination, the pathologic complete response rates were 19% to 53%, which were higher than those of the standard cisplatin plus fluorouracil regimen.^10-12^ These inspiring results have led to a prevalence in the use of paclitaxel-based regimens for dCRT in patients with esophageal cancer, without the evidence of phase III randomized clinical trials.

Schnirer et al^13^ from the MD Anderson Cancer Center first combined paclitaxel and fluorouracil in concurrent chemoradiotherapy for esophageal cancer, and they showed well-tolerated results. A small-sample-size trial, RTOG 0113, subsequently compared two paclitaxel-based regimens with the cisplatin plus fluorouracil regimen from the RTOG 9405 trial in dCRT for patients with localized esophageal cancer, and showed that the paclitaxel plus fluorouracil regimen had an increasing trend compared with the cisplatin plus fluorouracil regimen (1-year OS, 76% *v* 69%, *P* = .104), although the difference was not statistically significant.^14,15^ Subsequently, several single-arm phase II trials of the paclitaxel plus fluorouracil regimen used for patients with locally advanced and advanced esophageal cancer showed promising efficacy, with 3-year OS rates of 35.8% to 42.0%.^16,17^ High-quality data from prospective randomized controlled phase III trials are necessary to provide robust evidence for the efficacy of the paclitaxel-based regimen used in dCRT.

Considering these factors, we initiated ESO-Shanghai 1, a multicenter, randomized, open-label phase III study, in 2012 to investigate whether the paclitaxel plus fluorouracil regimen was superior in terms of 3-year OS to the standard cisplatin plus fluorouracil regimen in dCRT for patients with locally advanced esophageal squamous cell carcinoma (ESCC). The paclitaxel plus fluorouracil regimen used in this trial referred to the RTOG 0113 trial, with modifications because of the high toxicities in that trial.^15^

## PATIENTS AND METHODS

### Study Design

In the ESO-Shanghai 1 trial, we recruited patients in seven trial centers located in China (Appendix Table A1, online only) who met the following key eligibility criteria (for full inclusion and exclusion criteria, refer to Appendix Table A2, online only): histologically proven squamous cell esophageal carcinoma, stage IIA to IVa (American Joint Committee on Cancer, 6th edition), previously untreated; 18 to 75 years of age; Eastern Cooperative Oncology Group performance status of 2 or below; no severely abnormal hematopoietic, cardiac, pulmonary, renal, or hepatic function; and adequate hematologic function. The synopsis of the protocol for the study has been published elsewhere.^18^ The protocol was approved by the Ethics Committee of the Fudan University Shanghai Cancer Center (1203108-4). All participants provided written informed consent.

### Random Assignment

Eligible patients were randomly allocated at a 1:1 ratio to the cisplatin plus fluorouracil group or the paclitaxel plus fluorouracil group by a central randomization center (Fudan University Shanghai Cancer Center, Shanghai, China). Statistical analysis system 9.3 was used to generate a random permutation sequence and to produce patient random assignment numbers. Random assignment was stratified by the investigator centers, performed centrally by the statistician, and provided to the respective investigators via telephone.

### Treatment

Both groups received the same radiotherapy with photons (6 MV) to a total dose of 61.2 Gy in 34 fractions (5 days per week at 1.8 Gy/d) according to the treatment guideline of radiotherapy for Chinese esophageal carcinoma.^19^ The specific indications of chemotherapy in the cisplatin plus fluorouracil group were fluorouracil 1,800 mg/m^2^ continuous intravenous 72 h on day 1 and cisplatin 25 mg/m^2^/d on days 1 to 3 every 4 weeks for two cycles in concurrent chemotherapy and two cycles in consolidation chemotherapy. The specific indications of chemotherapy in the paclitaxel plus fluorouracil group were fluorouracil 300 mg/m^2^ continuous intravenous 96 h (initiated on day 1 and terminated on day 4) and paclitaxel 50 mg/m^2^/d on day 1 every week for five cycles in concurrent chemotherapy and fluorouracil 1,800 mg/m^2^ continuous intravenous 72 h (initiated on day 1 and terminated on day 3) with paclitaxel 175 mg/m^2^/d on day 1 every 4 weeks for two cycles in consolidation chemotherapy.

### Outcomes

The primary end point of this trial was 3-year OS. We defined OS as the time between the start of the study treatment (day 1) and death from any cause or last follow-up for patients alive at the end of the study. The secondary end points included progression-free survival (PFS), defined as the time between day 1 and the first event of local failure, metastatic recurrence, progression, or death, and the number and grade of participants with adverse events (AE).

### Statistical Analysis

We designed this trial to test the inferiority of 3-year OS in the paclitaxel plus fluorouracil group versus the cisplatin plus fluorouracil group. With a global alpha risk of 5% and 80% power, an accrual period of 48 months, a minimum follow-up of 36 months, and 6% patient loss, the inclusion of 436 patients (1:1 random assignment) would be necessary to demonstrate an improvement of 12% in OS at 3 years (from 30% in the cisplatin plus fluorouracil group to 42% in the paclitaxel plus fluorouracil group, on the basis of the results of the RTOG 8501 clinical trial and a phase II study).^3,16^ The study would be terminated when 293 events occurred or when the follow-up times of the surviving patients enrolled all surpassed 3 years. We did not plan to undertake interim analyses.

We used the Kaplan-Meier method to estimate the event time and to compare OS and PFS among the treatment arms with an unadjusted log-rank test on an intention- to-treat basis (including all patients who underwent random assignment). Cox regression was used to estimate the hazard ratios. Pearson’s χ^2^ or Fisher’s exact tests were used to compare between the two groups the toxicities and treatment compliance in the patients who received at least one cycle of chemotherapy. A *P* value of < .05 was used as the significance threshold. Data were analyzed with SPSS version 19.0 (SPSS, Chicago, IL). Detailed correlations of the AE with the radiation dose volume histogram in radiotherapy treatment delivery will be presented in future articles.

## RESULTS

**FIG 1.**
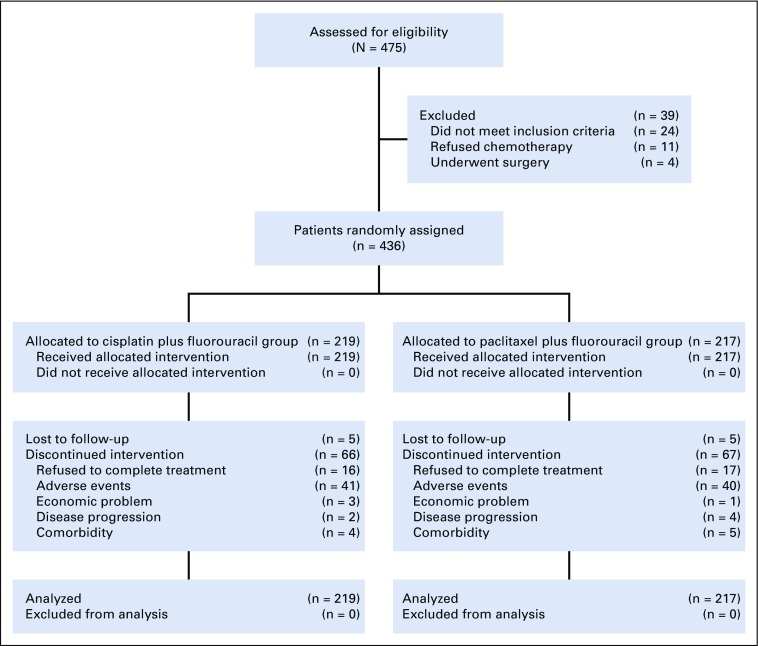
Trial profile. Four hundred seventy-five patients with esophageal squamous cell carcinoma were assessed for eligibility at registration in seven centers in China. Two hundred nineteen patients were assigned to the cisplatin plus fluorouracil group and 217 patients were assigned to the paclitaxel plus fluorouracil group as an intention-to-treat population.

Between April 2012 and July 2015, 436 patients with ESCC in six centers (Appendix Table A1) were randomly assigned (Fig 1). Both groups had acceptable completion rates, and the full treatment completion rates were similar between the paclitaxel plus fluorouracil group and the cisplatin plus fluorouracil group (138 of 217 [63.6%] *v* 152 of 219 [69.4%], respectively; *P* = .199). The baseline patient and tumor characteristics were well balanced between the two groups (Table 1). The median age was 62 years (interquartile range [IQR], 56-68 years) in the paclitaxel plus fluorouracil group and 62 years (IQR, 56-68 years) in the cisplatin plus fluorouracil group. We identified incorrect staging for supraclavicular lymph node metastasis in 42 patients (9.6%) after staging review. For this reason, 17 patients (7.8%) in the paclitaxel plus fluorouracil group and 25 patients (11.4%) in the cisplatin plus fluorouracil group who were staged IVa initially were upstaged to IVb. The median tumor lengths of the patients in the paclitaxel plus fluorouracil group and the cisplatin plus fluorouracil group were 6 cm (IQR, 4.5-7.5 cm) and 6 cm (IQR, 4.5-7.5 cm), respectively.

**TABLE 1.**
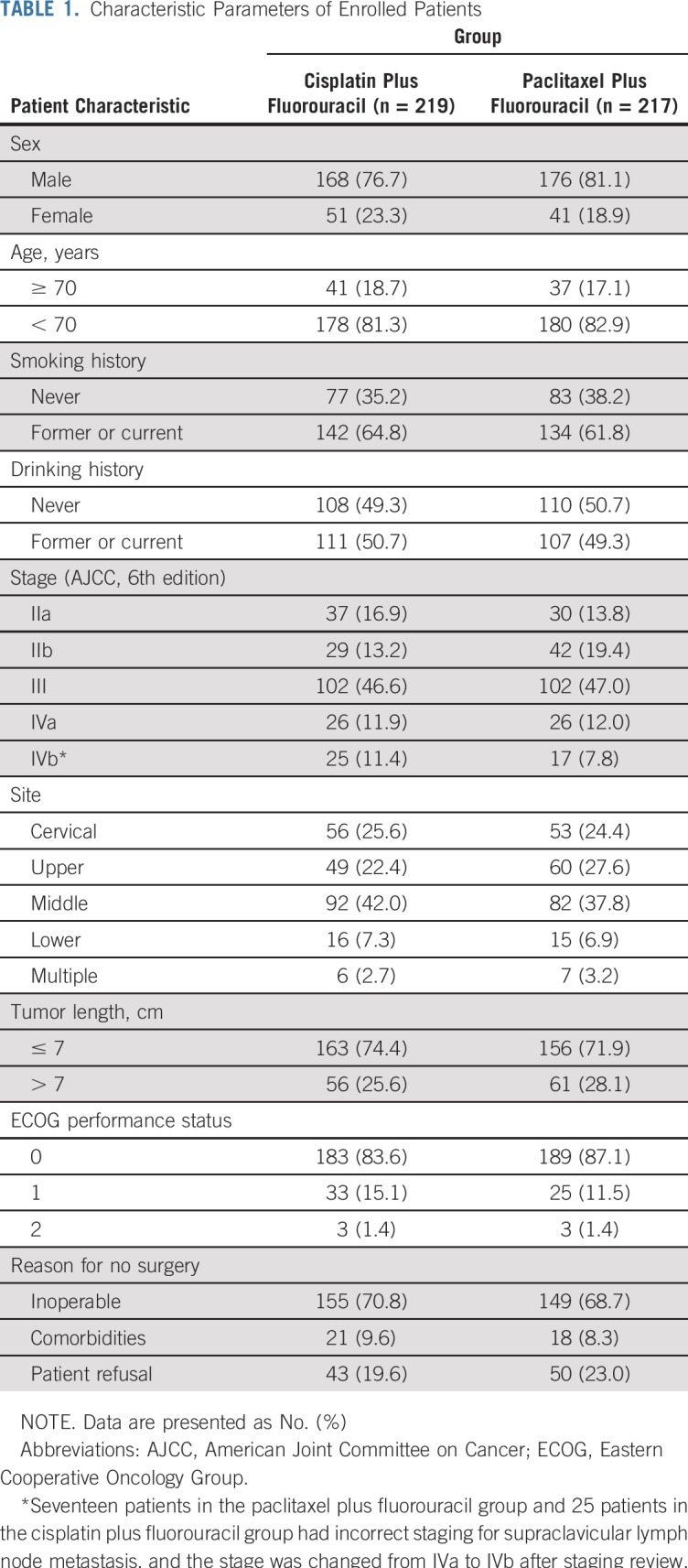
Characteristic Parameters of Enrolled Patients

Details of the chemotherapy compliance in the randomly assigned patients are listed in Appendix Table A3 (online only). All patients in the cisplatin plus fluorouracil group completed at least 50% of concurrent chemotherapy, compared with 212 patients (97.7%) in the paclitaxel plus fluorouracil group (*P* = .030). Similar numbers of patients in the paclitaxel plus fluorouracil group (172 [79.3%]) and the cisplatin plus fluorouracil group (172 [78.5%]) completed at least one cycle of consolidation chemotherapy (*P* = .853). At least one delay was reported in 123 patients (56.7%) in the paclitaxel plus fluorouracil group, compared with 92 patients (42.0%) in the cisplatin plus fluorouracil group (*P* = .002). Chemotherapy delay and cessation were mainly caused by treatment-induced toxicities.

Radiotherapy parameters and compliance for the two groups are detailed in Appendix Table A4 (online only). The tumor volume and dose volume histogram parameters of the lung and heart were well balanced between the two groups. Two hundred ten patients (96.8%) in the paclitaxel plus fluorouracil group and 213 patients (97.3%) in the cisplatin plus fluorouracil group completed at least 50 Gy radiotherapy. The main reason for the premature cessation of radiotherapy was treatment-induced toxicities. There was no significant difference between the two groups in the total delay of the radiotherapy delivery time.

**FIG 2.**
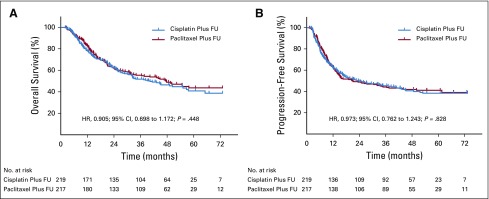
(A) Overall survival and (B) progression-free survival in enrolled patients. There was no significant difference between the paclitaxel plus fluorouracil group and the cisplatin plus fluorouracil group in terms of overall survival or progression-free survival. HR, hazard ratio.

**FIG 3.**
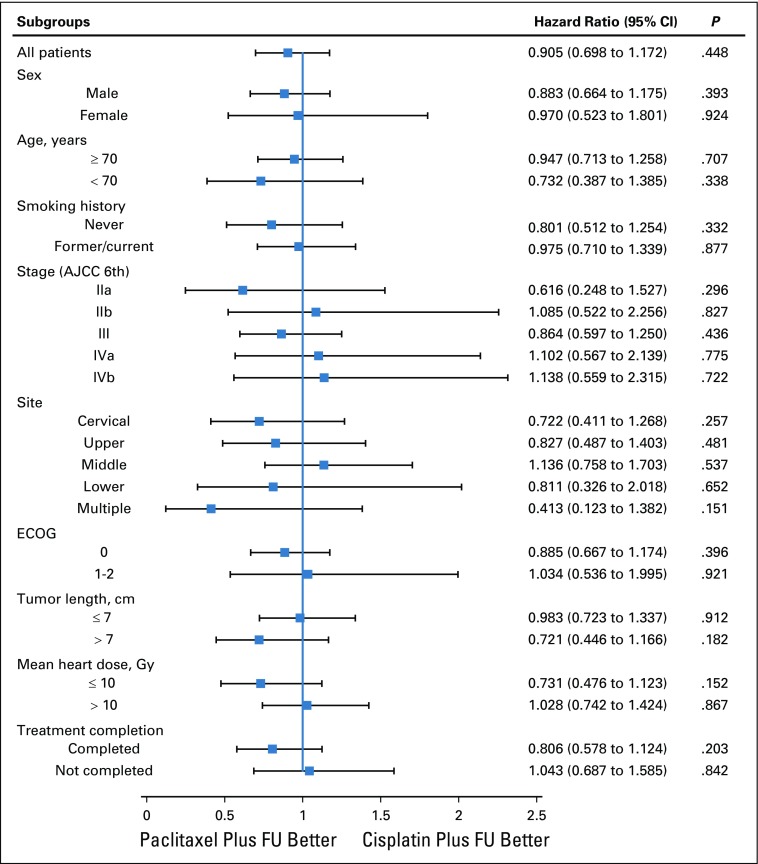
Subgroup analyses of overall survival. The effects of different regimens on overall survival according to the predictive and prognostic factors (sex, age, smoking history, stage, tumor site, Eastern Cooperative Oncology Group (ECOG), tumor length, mean heart dose, and treatment completion) were not significantly different between the two groups. AJCC, American Joint Committee on Cancer.

At the analysis time of Aug 1, 2018, the median follow-up of the surviving patients was 48.7 months (IQR, 42.6-60.9 months) for intention to treat (48.7 months [IQR, 42.6-60.9 months] in the paclitaxel plus fluorouracil group and 54.7 months [IQR, 42.6-60.9 months] in the cisplatin plus fluorouracil group). Two hundred thirty deaths (52.8%) were recorded, including 110 deaths (50.7%) in the patients allocated to the paclitaxel plus fluorouracil group and 120 deaths (54.8%) in the patients allocated to the cisplatin plus fluorouracil group. There was no significant difference in 3-year OS (55.4% *v* 51.8%, respectively; hazard ratio, 0.905 [95% CI, 0.698 to 1.172]; *P* = .448) or median survival (47.6 months *v* 40.3 months, respectively) between the paclitaxel plus fluorouracil group and the cisplatin plus fluorouracil group, respectively (Fig 2). The 1, 2, and 5-year OS rates were 79.3%, 60.6%, and 44.3%, respectively, in the paclitaxel plus fluorouracil group and 76.2%, 61.5%, and 40.8%, respectively, in the cisplatin plus fluorouracil group. This pattern was consistent across the relevant predictive and prognostic factors (Fig 3).

**FIG A1.**
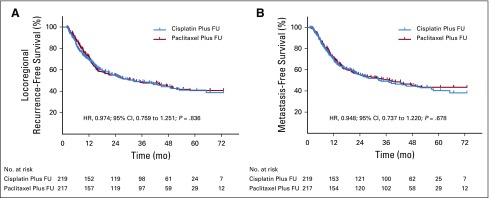
(A) Locoregional progression-free survival and (B) metastasis-free survival in enrolled patients. Differences between the paclitaxel plus fluorouracil group and the cisplatin plus fluorouracil group in terms of locoregional progression-free survival (locoregional progression included recurrences at the primary tumor and regional lymph node) and metastasis-free survival (metastases included any site beyond the primary tumor and regional lymph nodes) were not significant. FU, fluorouracil; HR, hazard ratio.

Overall, at the analysis time of Aug 1, 2018, 178 patients (40.8%) were alive without disease progression, with 90 patients (41.5%) in the paclitaxel plus fluorouracil group and 88 patients (40.2%) in the cisplatin plus fluorouracil group. Median PFS was 21.0 months (95% CI, 8.7 to 33.3 months) in the paclitaxel plus fluorouracil group and 24.3 months (95% CI, 10.9 to 37.7 months) in the cisplatin plus fluorouracil group. No significant differences were identified in 3-year PFS (43.7% *v* 45.5%, respectively; hazard ratio, 0.973 [95% CI, 0.762 to 1.243]; *P* = .828) between the paclitaxel plus fluorouracil and the cisplatin plus fluorouracil group (Fig 2). Moreover, the differences between the two groups in terms of locoregional recurrence-free survival and metastasis-free survival were not significant (Appendix Fig A1, online only). The patterns of treatment failure in each group are listed in Appendix Table A5 (online only).

Because all randomly assigned patients received at least one cycle of chemotherapy, the safety population in this trial was equal to the intention-to-treat population. All grade 3 or higher AE and grade 1 to 2 AE that occurred in more than 10% of patients reported during treatment are listed in Table 2. There was no significant difference between the two groups in the incidence of acute grade 3 or higher AE (106 [48.8%] in the paclitaxel plus fluorouracil group *v* 113 [51.6%] in the cisplatin plus fluorouracil group, respectively, *P* = .566). The paclitaxel plus fluorouracil group had significantly lower incidences of acute grade 3 or higher anemia (six [2.8%] *v* 16 [7.3%], respectively; *P* = .030), thrombocytopenia (one [0.5%] *v* 33 [15.1%], respectively; *P* = .000), anorexia (three [1.4%] *v* 33 [15.1%], respectively; *P* = .000), nausea (three [1.4%] *v* 32 [14.6%], respectively; *P* = .000), vomiting (five [2.3%] *v* 41 [18.7%], respectively; *P* = .000), and fatigue (15 [6.9%] *v* 46 [21.0%], respectively; *P* = .000) and significantly higher incidences of acute grade 3 or higher leukopenia (68 [31.3%] *v* 40 [18.3%], respectively; *P* = .002), radiation dermatitis (11 [5.1%] *v* three [1.4%], respectively; *P* = .032), and radiation pneumonitis (19 [8.8%] *v* six [2.7%], respectively; *P* = .007) than the cisplatin plus fluorouracil group. Long-term AE are listed in Table 3. Late cardiac disorders and late radiation pneumonitis were similar between the two groups. Although the patients in the paclitaxel plus fluorouracil group had a significantly higher incidence of grade 1 or higher late esophagitis than did the patients in the cisplatin plus fluorouracil group (28 [12.9%] *v* 11 [5.0%], respectively; *P* = .004), there was no significant difference in the patients who had grade 2 or higher late esophagitis between the two groups.

**TABLE 2.**
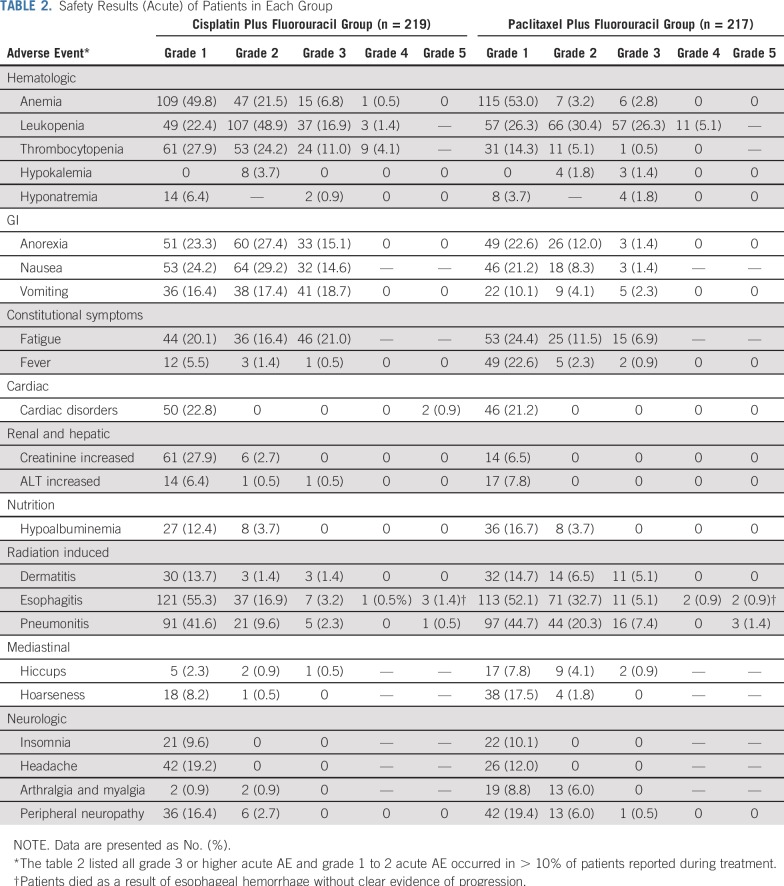
Safety Results (Acute) of Patients in Each Group

**TABLE 3.**
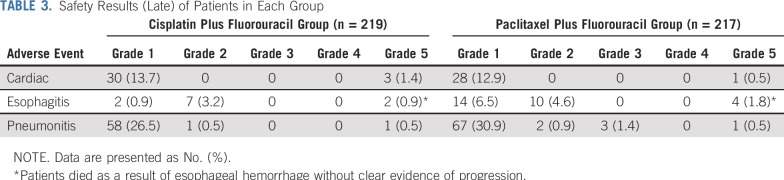
Safety Results (Late) of Patients in Each Group

## DISCUSSION

For several decades, paclitaxel-based regimens have been widely used in concurrent chemoradiotherapy in patients with inoperable esophageal cancer in routine clinical practice and in trials worldwide, despite the lack of level 1 evidence.^7-9,20^ To our knowledge, our trial is the first multicenter, randomized, phase III trial to compare the paclitaxel-based regimen with the cisplatin plus fluorouracil regimen in dCRT in patients with locally advanced ESCC. Our findings showed that dCRT with paclitaxel plus fluorouracil was not superior to cisplatin plus fluorouracil, whereas the AE profiles of the two regimens were different. On the basis of our results, we suggest that the cisplatin plus fluorouracil regimen remain the standard regimen in dCRT for patients with locally advanced ESCC.

Although one half of the patients enrolled were stage III or IV, the 3-year OS rates in both groups in our trial (55.4% in the paclitaxel plus fluorouracil group and 51.8% in the cisplatin plus fluorouracil group) were higher than in the RTOG 8501 and PRODIGE5/ACCORD17 trials (19.9% to 30.0%).^3,21^ The full chemotherapy compliance rate in our trial was substantially higher (65% in the paclitaxel plus fluorouracil group and 69% in the cisplatin plus fluorouracil group) than in the RTOG 8501 trial (54% in combined therapy with cisplatin plus fluorouracil) and was similar to that of the PRODIGE5/ACCORD17 trial (71% in the FOLFOX group and 76% in the cisplatin plus fluorouracil group). Reasons likely included improvements in radiotherapy techniques, staging methods, and best supportive care. We used involved field irradiation with intensity modulated radiation therapy to decrease the toxicities of normal tissues, pragmatically reduced the dose of fluorouracil, and split the administration of cisplatin into 3 days on the basis of the experiences of our institute to reduce chemotherapy toxicities.^22,23^ Furthermore, ethnic differences may have played an important role.^24,25^

Our findings showed that the AE profiles significantly differed between the two regimens. The incidences of severe acute GI toxicities and thrombocytopenia in the cisplatin plus fluorouracil group were approximately 10 times to 30 times those of the paclitaxel plus fluorouracil group, respectively (anorexia, 15.1% *v* 1.4%; nausea, 14.6% *v* 1.4%; vomiting, 18.7% *v* 2.3%; and thrombocytopenia, 15.1% *v* 0.5%). Moreover, severe anemia was also higher in the cisplatin plus fluorouracil group than in the paclitaxel plus fluorouracil group (7.3% *v* 2.8%, respectively). In contrast, although the paclitaxel plus fluorouracil group showed significantly higher incidences of severe acute leukopenia, radiation-induced dermatitis and radiation pneumonitis compared with the cisplatin plus fluorouracil group, the incidence of each severe nonhematologic AE in the paclitaxel plus fluorouracil group was under 9%.

There has been continued controversy over whether the paclitaxel-based regimen would enhance the risk of radiation pneumonitis when combined with radiotherapy. A systematic review and Veterans’ Health Administration data in the United States showed that carboplatin plus paclitaxel compared with etoposide plus cisplatin did not increase the risk of radiation pneumonitis in dCRT for stage III non–small-cell lung cancer.^26,27^ However, retrospective studies have indicated that paclitaxel-based dCRT did significantly increase the risk of radiation pneumonitis, with an odds ratio of 3.33.^28,29^ Liang et al^30^ recently published a phase III trial that compared etoposide plus cisplatin with carboplatin plus paclitaxel in dCRT for patients with stage III non–small-cell lung cancer. The results showed that the incidence of grade 2 or higher radiation pneumonitis was significantly higher in the carboplatin plus paclitaxel arm than in the etoposide plus cisplatin arm (33.3% *v* 18.9%, respectively; *P* = .036); however, the incidence of grade 4 or 5 radiation pneumonitis was not significantly different (5.2% *v* 4.2%, respectively). In our trial, we observed similar results, with a significantly higher incidence of grade 2 or 3 acute radiation pneumonitis and similar incidences of grade 4 or 5 acute radiation pneumonitis and late radiation pneumonitis. In view of the consistent results of the two phase III studies, both of which had well-balanced baselines of the lung parameters of dose-volume histogram, we suggest that the paclitaxel-based regimen only increased the risk of grade 2 to 3 radiation pneumonitis and did not increase grade 4 to 5 acute radiation pneumonitis or any grade of late radiation pneumonitis when combined with thoracic radiotherapy.

Several limitations should be considered when interpreting our findings. First, we may have underestimated the efficacy of the standard cisplatin plus fluorouracil regimen. The 3-year OS of the cisplatin plus fluorouracil regimen was substantially higher in our trial than in the RTOG 8501 trial (51% *v* 30%, respectively).^3^ Therefore, it may not be appropriate to use the historical data of the RTOG 8501 trial, which was conducted decades ago, to estimate the 3-year OS of the standard cisplatin plus fluorouracil group in our trial and calculate the sample size. We overestimated the expected change of the 3-year OS between the paclitaxel plus fluorouracil group and cisplatin plus fluorouracil group and did not design for a noninferiority comparison. Second, we did not assess the quality of life. We may find a difference in the quality of life between the two groups because the cisplatin plus fluorouracil regimen showed a significantly more frequent incidence of severe GI toxicities than did the paclitaxel plus fluorouracil regimen in our trial. Third, we did not compare different paclitaxel-based regimens or optimize the dosage of the paclitaxel plus fluorouracil regimen before this trial. Because of this, we are launching a comparison of paclitaxel plus cisplatin, paclitaxel plus carboplatin, and paclitaxel plus fluorouracil concurrent with radiotherapy for patients with ESCC (ESO-Shanghai 2) in China as a multicenter randomized phase III trial (ClinicalTrials.gov identifier: NCT02459457) to clarify the optimal paclitaxel-based regimen.

In conclusion, we failed to confirm that the paclitaxel plus fluorouracil regimen was superior in terms of OS to the standard cisplatin plus fluorouracil regimen in the dCRT for patients with ESCC. The cisplatin plus fluorouracil regimen remained the standard regimen in dCRT for patients with locally advanced ESCC. In addition, when we compared the different AE profiles between the two regimens, we found that the paclitaxel plus fluorouracil regimen had higher incidences of severe leukopenia, radiation dermatitis, and radiation pneumonitis and lower incidences of anemia, thrombocytopenia, GI toxicities, and fatigue.

## Figures and Tables

**TABLE A1.**
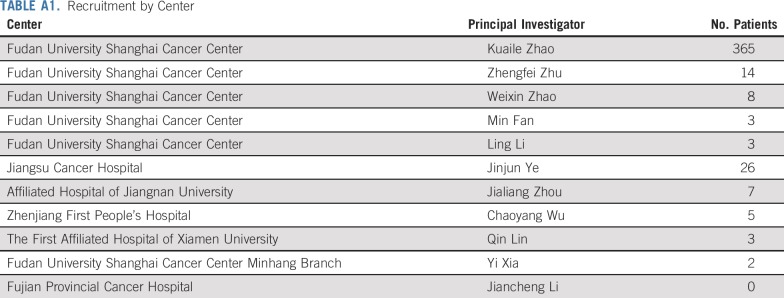
Recruitment by Center

**TABLE A2.**
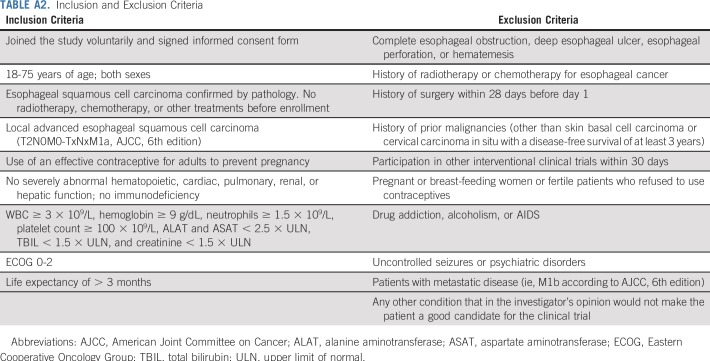
Inclusion and Exclusion Criteria

**TABLE A3.**
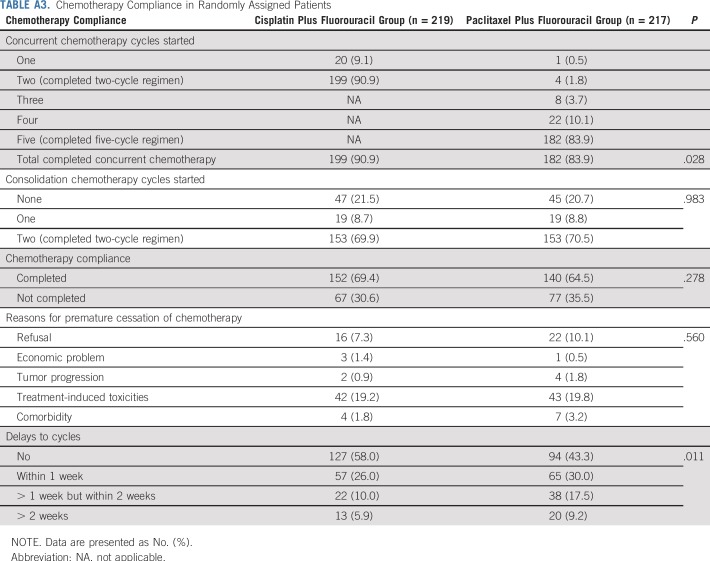
Chemotherapy Compliance in Randomly Assigned Patients

**TABLE A4.**
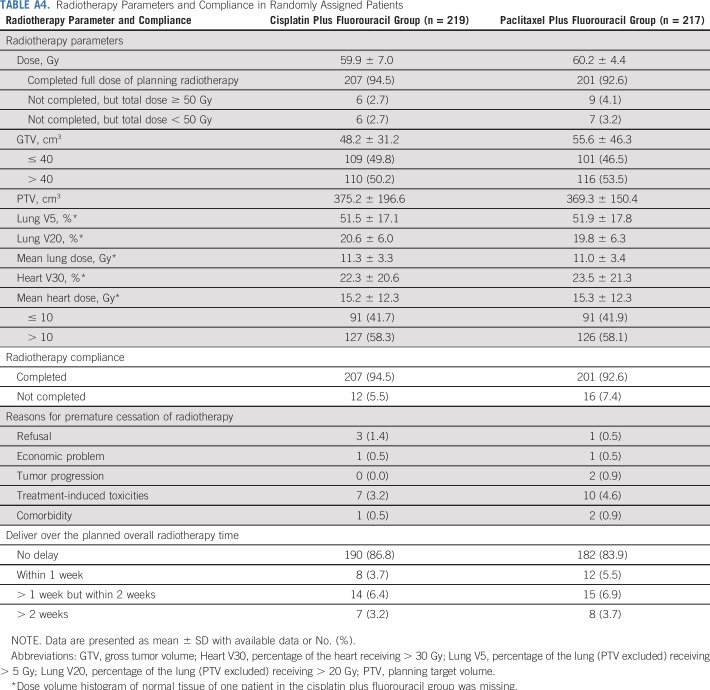
Radiotherapy Parameters and Compliance in Randomly Assigned Patients

**TABLE A5.**
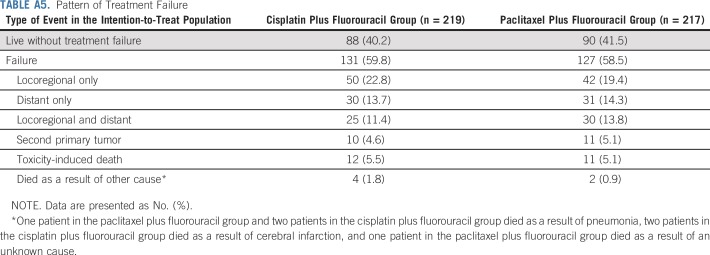
Pattern of Treatment Failure
